# Excretory/Secretory Products from *Trichinella spiralis* Adult Worms Ameliorate DSS-Induced Colitis in Mice

**DOI:** 10.1371/journal.pone.0096454

**Published:** 2014-05-02

**Authors:** Xiaodi Yang, Yaping Yang, Yunyun Wang, Bin Zhan, Yuan Gu, Yuli Cheng, Xinping Zhu

**Affiliations:** 1 Department of Parasitology, School of Basic Medical Sciences, Capital Medical University, Beijing, China; 2 Department of Microbiology and Parasitology, Bengbu Medical College; Anhui Key Laboratory of Infection and Immunity, Bengbu, Anhui, China; 3 Section of Tropical Medicine, Department of Pediatrics, Baylor College of Medicine, Houston, Texas, United States of America; University of South Carolina School of Medicine, United States of America

## Abstract

**Background:**

Many evidences show the inverse correlation between helminth infection and allergic or autoimmune diseases. Identification and characterization of the active helminth-derived products responsible for the beneficial effects on allergic or inflammatory diseases will provide another feasible approach to treat these diseases.

**Methods and Findings:**

Colitis was induced in C57BL/6 mice by giving 3% DSS orally for 7 days. During this period, the mice were treated daily with the excretory/secretory products from *T. spiralis* adult worms (AES) intraperitoneally. The severity of colitis was monitored by measuring body weight, stool consistency or bleeding, colon length and inflammation. To determine the *T. spiralis* AES product-induced immunological response, Th1, Th2, Th17 and regulatory cytokine profiles were measured in lymphocytes isolated from colon, mesenteric lymph nodes (MLN), and the spleen of treated mice. The CD4^+^ CD25^+^ FOXP3^+^ regulatory T cells (Tregs) were also measured in the spleens and MLN of treated mice. Mice treated with AES significantly ameliorated the severity of the DSS-induced colitis indicated by the reduced disease manifestations, improved macroscopic and microscopic inflammation correlated with the up-regulation of Treg response (increased regulatory cytokines IL-10, TGF-beta and regulatory T cells) and down-regulation of pro-inflammatory cytokines (IFN-gamma, IL-6 and IL-17) in the spleens, MLN and colon of treated mice.

**Conclusions:**

Our results provide direct evidences that *T. spiralis* AES have a therapeutic potential for alleviating inflammatory colitis in mice. This effect is possibly mediated by the immunomodulation of regulatory T cells to produce regulatory and anti-inflammatory cytokines and inhibit pro-inflammatory cytokines.

## Introduction

Inflammatory bowel diseases (IBD), including Crohn's disease (CD) and ulcerative colitis (UC), are chronic and relapsing inflammatory conditions of the gastrointestinal tract. The etiology and pathogenesis of these diseases have not been definitively elucidated; however, a complex interplay of genetic, microbial and environmental factors has been considered to be attributable for the abnormal immune responses and subsequent intestinal inflammation [1], [2], [3].

More than 20 years ago, the *hygiene hypothesis* was proposed by Strachan, claiming a relationship between the increase in allergic and autoimmune diseases and the lack of exposure to helminth and other infections [4]. An increasing amount of epidemiological evidence has revealed the inverse association of autoimmune or allergic diseases with helminth infections [5], [6], [7]. Since then, a significant number of experimental studies have provided support for this hypothesis and demonstrated that certain helminths, including *Trichinella spiralis*, have immunomodulatory effects not only on parasite-induced inflammation but also on other immuno-pathologies, such as allergies and autoimmune diseases [8], [9], [10], [11], [12], [13]. Subsequently, early clinical trials for IBD patients with whipworm *Trichuris muris* or hookworm *Necator americanus* infections have demonstrated an amelioration of symptoms with a decreased disease activity [14], [15], [16], [17], [18]. Although not fully clarified, the possible mechanism that underlies the hygiene hypothesis is the immunomodulatory effects of the molecules secreted by parasitic helminthes during parasitism on the host immune response as a strategy to evade host immune attack. These immunomodulations include a strong Th2 immune response and/or regulatory cytokines (IL-10, TGF-β) and T-regulatory (Treg) response to down-regulate the cellular responses to the parasites. As a result, this down-regulation of cellular response could reduce the host's excessive pro-inflammatory responses to some autoimmune or allergic diseases [19], [20], [21], [22].

The immunomodulatory effects of helminth infection raise the intriguing strategy of using the ancient helminth infections to treat modern autoimmune or allergic diseases [23]. However, helminth infections in humans could also lead to pathology and disease. Direct treatment with the living worm is widely unacceptable ethically and physically. Therefore, the proteins secreted by parasitic helminthes involved in the immunomodulation have become more attractive targets as a safe substitute for living parasite infections for autoimmune therapies [24].

Ruyssers et al. [25] and Cancado et al. [26] recently demonstrated the therapeutic potential of excretory/secretory (ES) products from adult hookworms, *Ancylostoma caninum* and *A. ceylanicum*, on experimental colitis in mice, predominantly through the down-regulation of Th1 and Th17 cytokines. Although helminthic ES products could directly modulate dendritic cells (DC), suppress the expression of co-stimulatory MHCII and produce anti-inflammatory cytokines [20], modulation of the immune system by ES products derived from different helminth species or developmental stages may act differently [27], [28], [29], [30]. *T. spiralis* is one of the most widespread zoonotic parasitic nematodes in the world. Its life cycle is completed in a single host and includes three stages: muscle larvae inside skeletal striated muscle cells, adult worms in the small intestine, and newborn larvae in the lymphatic vessels and bloodstream. During different phases or stages of parasite growth, *T. spiralis* interacts with the host immune system to evade or inhibit the host immune response through releasing a number of proteins into its surrounding environment, which are considered to be crucial for its successful invasion and survival within the host [31], [32], [33], [34]. Several studies have recently demonstrated that *Trichinella* secreted proteins or infection itself are able to induce a strong Th2/Treg response and the production of Th2/immunoregulation cytokines, e.g., IL-4, IL-5, IL-10, IL-13, and TGF-β [29], [32], [35], [36], [37], which are associated with the amelioration of autoimmune diseases such as colitis [38], [39], allergic airway inflammation [40], [41], Type I diabetes [42] and autoimmune encephalomyelitis [43].

In this study, we further explored the therapeutic effects of ES products from *T. spiralis* adult worms (AES) on DSS-induced colitis in C57BL/6 mice. The goal of this study is to better understand the possible mechanisms behind their treatment efficacy as a potential strategy to develop therapeutics for incurable immune disorders using products derived from helminths, which have lived within humans for millions of years.

## Materials and Methods

### Ethics statement

All experimental animals were purchased from Laboratory Animal Services Center of Capital Medical University (Beijing, China). All experimental procedures were reviewed and approved by the Capital Medical University Animal Care and Use Committee and were consistent with the NIH Guidelines for the Care and Use of Laboratory Animals.

### Animals and DSS-induced colitis

Female C57BL/6 mice, aged 6–8 weeks and free of specific pathogens, were provided by the Laboratory Animal Center, Academy of Military Medical Sciences.

The colitis was induced in C57BL/6 mice with dextran sodium sulfate (DSS) as previously described [44]. Briefly, DSS (40 kDa, Applichem, Germen) was dissolved in sterile filtered water at a final concentration of 3% and presented to mice as drinking water for 7 consecutive days. Freshly made DSS solution was provided daily in drinking water. Negative control animals received filtered water only. During the colitis induction, all mice were treated with *Trichinella* AES as described below. On the 8^th^ day, all animals were sacrificed at the end of experiments by cervical dislocation.

### Preparation of parasites and excretory/secretory products from adults (AES)

*T. spiralis* (strain ISS 533) was maintained in female ICR mice. Muscle larvae were recovered from the muscles of infected mice using a standard pepsin/hydrochloric acid digestion method [45]. Adult *T. spiralis* worms were obtained from the intestine of a rat orally infected with 12,000 muscle larvae after 84 hours [46]. *T. spiralis* AES were prepared and collected as previously described [29]. Briefly, *T. spiralis* adult worms recovered from intestine of infected rat were washed three times with phosphate-buffered saline (PBS) and then cultured in RPMI-1640 medium supplemented with 100 U/ml penicillin, 100 U/ml streptomycin and 0.25 µg/ml amphotericin B at 37°C and 5% CO_2_ for 48 hours. The culture supernatant was collected by centrifugation, then filtered through a 0.45 micron syringe filter and buffer exchanged into PBS. The protein concentrations of the prepared ES products were determined using BCA assay (Pierce, USA).

### Experimental design

In the first experiment, we investigated the effect of *T. spiralis* adult ES products on inhibiting the development of DSS-induced colitis in mice. Briefly, the mice were divided into 4 groups (12–16 mice each group). The first two groups of mice were induced with DSS to develop colitis as described above. Concurrently, each group of mice was intraperitoneally injected daily with 25 µg of AES (DSS-AES) or PBS only as a control (DSS-PBS) in a total volume of 100 µl for 7 days of concomitant DSS-colitis induction. The other two groups of mice were treated with the same amount of AES or PBS for 7 days without colitis induction as a control (AES-control and PBS-control, respectively).

After seven days of treatment, the mice were sacrificed, their spleens, mesenteric lymph nodes (MLNs), and colon were aseptically removed and the cells were isolated for cell culture for cytokine profiling. Their entire large intestines were collected for measuring the pathology and cytokine profile. The pathology of each intestine was evaluated based on 5 criteria described in detail as below: clinical disease activity index, the length of the colon, macroscopic score, microscopic inflammation score and myeloperoxidase (MPO) activity. Except for the principal investigator, all investigators were blind to all solution contents and mice groups until the end of the experiments.

### Clinical disease score

The mice were observed daily for morbidity and given a clinical disease score (disease activity index, DAI) between 0 and 12 based on the following characteristic criteria: weight loss, diarrhea, and bleeding feces [44] (Table 1).

**Table 1:** The criteria for scoring the DSS-induced colitis (DAI).

Pathologic Score	0	1	2	3	4
Weight loss	none	1–5%	5–10%	10–15%	>15%
Stool shape	normal	between	loose stool	between	watery diarrhea
Stool bleeding	none	between	slight bleeding	between	gross bleeding

### Macroscopic inflammation score

After being sacrificed, the mouse colon was removed and opened longitudinally, and the colonic damage was assessed macroscopically. Briefly, four parameters were taken into account: presence of adhesions, degree of colonic ulcerations, wall thickness, and degree of mucosal edema. Each parameter was given a score from 0 (normal) to 3 (severe) as previously described [47]. The total score ranged from a minimum of 0 to a maximum of 12. The length of colon as a way to evaluate the extent of inflammation [51] was also measured.

### Microscopic inflammation score

Small segments of the colon taken for histopathology examination were fixed in 4% neutral-buffered formalin, embedded into paraffin, sectioned at 5 µm thickness and stained with hematoxylin and eosin. To evaluate the severity of inflammation, we adopted the histological damage score from Obermeier et al. [48] based on the following parameters: (a) Epithelial damage (0 point = none, 1 point = minimal loss of goblet cells, 2 points = extensive loss of goblet cells, 3 points = minimal loss of crypts and extensive loss of goblet cells, and 4 points = extensive loss of crypts), (b) Infiltration (0 point = none, 1 point =  infiltration around crypt bases, 2 points =  infiltration in the muscularis mucosa, 3 points = extensive infiltration in the muscularis mucosa with edema, and 4 points = infiltration of the submucosa). The total score ranged from a minimum of 0 to a maximum of 8.

### MPO activity assay

Myeloperoxidase (MPO) activity, an enzyme occurring nearly exclusively in neutrophils, was determined using a MPO assay kit (Nanjing Jiancheng Bio-engineering Institute, China). Briefly, 100 mg of colon tissue was cut and homogenized in 1.9 ml of 50 mM PBS, pH 6.0, containing 0.5% hexadecyltrimethyl ammonium hydroxide and centrifuged at 12,000 rpm (4°C) for 20 min. The protein concentration of the colon extract supernatant was measured using a BCA protein assay kit (Thermo Scientific, USA). One hundred microliters of the supernatant was transferred into 2.9 ml of PBS (pH 6.0) containing 0.17 mg/ml 3,3′-dimethoxybenzidine and 0.0005% H_2_O_2_. The MPO activity of the supernatant was determined by measuring the H_2_O_2_-dependent oxidation of 3,3′-dimethoxybenzidine and is expressed as units per gram of total protein (U/g).

### Lymphocyte isolation and multiplex cytokine profiling

Spleens and MLNs were removed aseptically from the experimental mice, and the cells were isolated and suspended in RPMI-1640 supplemented with 5% bovine fetal serum, 100 mM L-glutamine, 100 U/ml penicillin, and 100 mg/ml streptomycin. Colons were collected and thoroughly washed with PBS and then cut into 0.5 cm pieces. The epithelia were removed by incubation with 1 mM DTT (Sigma-Aldrich, USA) and 1 mM EDTA (Sigma-Aldrich, USA) in RPMI 1640 medium supplemented with 5% FCS at 37°C for 20 min with gentle shaking. After repeating this step twice, the tissue was cut into smaller pieces and then incubated for 40 min at 37°C in 20 ml of RPMI 1640 containing 25 mM HEPES, 2 mM L-glutamine, 1 mM sodium pyruvate, 100 U/ml penicillin, 10 mg/ml gentamicin, 100 mg/ml streptomycin, and 1 mg/ml Liberase Research Grade Purified Enzymes (Roche, Mannheim, Germany). The cells suspension was filtered through a 100-µm filter and washed, and the lamina propria mononuclear cells (LPMC) were harvested by discontinuous 30/70% percoll gradient centrifugation.

The cells were subjected to a cytokine assay using IFN-γ, IL-4, IL-6, IL-10 (BD Biosciences, USA) and IL-17 (R&D Systems, USA) ELISPOT sets according to the manufacturer's instructions. Briefly, a total of 1×10^6^ cells were added to each well of 96-well MultiScreen HTS Filter plates (Millipore, USA) pre-coated with anti-mouse IL-4, IL-6, and IL-10, whereas 2×10^5^ cells were added to plates pre-coated with anti-IFN-γ and IL-17 antibodies. After being stimulated with anti-CD3 and anti-CD28 (BD Biosciences, USA) at a concentration of 1 µg/ml for 48 h (37°C), the cells were incubated with biotin-conjugated secondary antibodies and streptavidin-HRP (for the IFN-γ, IL-4, IL-6 and IL-10 assay). Single cytokine-positive cells were visualized by adding an AEC substrate and counted using an ELISPOT reader (CTL, USA) with the Immunospot image analyzer software (version 4.0). For the IL-17 assay, streptavidin-AP and BCIP/NBT chromogen were used [49], [50].

In addition, the levels of IL-13 and TGF-β in the cell culture medium were determined using ELISA kits following the manufacturer's instructions (R&D Systems, USA). Briefly, in which TGF-β was measured, cells (1×10^6^) were switched to serum-free media with 4 changes in media over a 24-hour period to reduce the background level of TGF-β provided by the cell culture serum. Samples were collected after being cultured for 24 hours past the last medium change, treated with 1 N HCl and then neutralized with 1.2 N NaOH/0.5 M HEPES to activate latent TGF-β. Hence, the presented data reflect the total TGF-β in the samples.

### Fluorescence-activated cell sorting (FACS) analysis of CD4^+^CD25^+^FOXP3^+^Tregs in the spleen and MLN lymphocytes

To evaluate the Tregs induced by treatment with the *T. spiralis* ES products, the isolated cells from the spleens and MLN of the treated mice were sorted by FACS using reagents from BD Pharmingen (USA). The cell surfaces were blocked with rat anti-mouse CD16/CD32 mAb for 15 min (4°C) and then incubated with anti-mouse CD3e, CD4, or CD25 mAbs or their isotype controls for surface marker staining. Subsequently, the cells were treated with FACS lysing solution for 10 min. After washing, fixation, permeabilization and a second blocking, the intracellular labeling of the Foxp3 protein was performed by treating the cells with anti-mouse Foxp3 mAb for 30 min on ice in the dark. The cells were washed twice and resuspended in 200 ml PBS containing 1% FBS followed by immediate flow cytometric analysis (BD Biosciences, USA).

### Statistical analyses

All data are presented as the mean ± standard error of the mean (SEM) and evaluated using a one-way ANOVA analysis and SPSS 11.5 software. *P<0.05* was regarded as statistically significant. All statistical analyses were performed using GraphPad Prism software.

## Results

### Effect of *T. spiralis* AES on relieving the severity of the DSS-induced acute colitis

**Figure 1:**
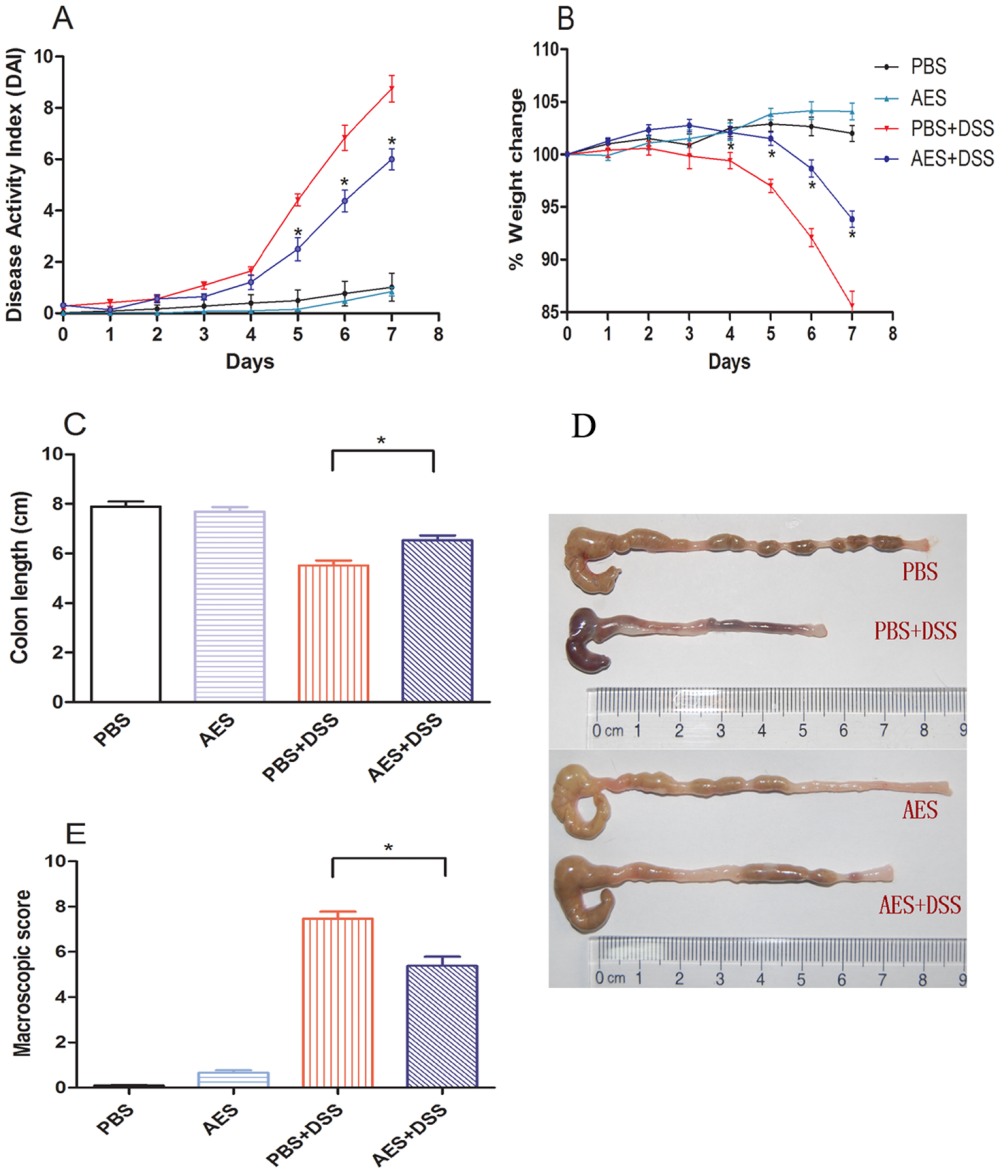
Treatment with *T. spiralis* AES ameliorated DSS-induced acute colitis in the C57BL/6 mice. (A) Change in the disease activity index (DAI) during the experimental period. (B) Changes in the percent body weight. (C, D) The colons were removed, and the length was measured. Representative colons are shown. (E) Colon macroscopic damage score based on the presence of adhesions, degree of colonic ulcerations, wall thickness, and degree of mucosal edema. The data are presented as the mean ± SE. Significant differences are indicated in each graph with asterisks (*P<0.05); n = 12 (number of mice included in each group).

After 7 days of oral DSS administration, all mice developed significant colitis manifestations such as weight loss, diarrhea, rectal bleeding, and physical weakness compared to the control mice treated with PBS only. The intraperitoneal treatment with 25 µg of AES significantly reduced the clinical score for the DSS-induced colitis in the mice (DSS-AES) compared to the PBS control (DSS-PBS) (Fig. 1, *P<0.05*). These improvements included lower disease activity indices (DAI, Fig. 1A), less body weight loss (Fig. 1B), less diarrhea or bleeding diarrhea and less colon shrinking (Fig. 1C, D, E). The shrinking or shortening of the colon and bleeding inside the colon were identified as the major signs of colitis induced by DSS [51]. Apparently, there was no DSS-induced bleeding or significant colon shrinking observed in mice treated with AES compared to PBS control mice (Fig. 1D). Similar to the PBS control, *T. spiralis* AES itself did not lead to obvious pathology to the treated mice except for minor intestinal edema at the intraperitoneal injection site (Fig. 1).

**Figure 2:**
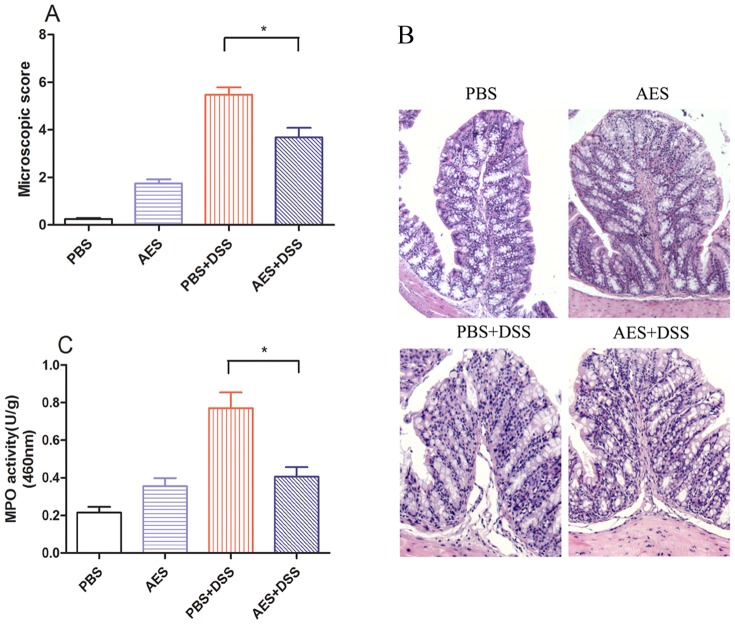
Treatment with *T. spiralis* AES reduced microscopic colon damage and the myeloperoxidase (MPO) activity induced by DSS in the C57BL/6 mice. (A) AES treatment decreased the microscopic damage score for the DSS-induced mouse colitis. (B) AES treatment reduced the DSS-induced epithelial destruction, edema, and infiltration of inflammatory cells on the colon histological sections. (C) MPO activity in the DSS-induced colon was reduced by treatment with AES. The data are presented as the mean ± SE. The asterisks* indicate statistical significance at *P<0.05*; n = 12 (number of mice included in each group).

DSS induction resulted in the loss of normal colonic architecture, microscopically characterized by marked epithelial destruction, edema, ulcerations, goblet cell depletion, and intense inflammatory infiltration (PBS+DSS) compared to the non-DSS-treated control (PBS) (Fig. 2A, B). However, treatment with AES significantly decreased the microscopic inflammation score for DSS-induced colitis (AES+DSS) compared to the PBS control (*P<0.05*). After being treated with *T. spiralis* AES, the colon MPO activity returned to a level similar to the control groups without DSS induction (*P<0.05*) (Fig. 2C), indicating that *T. spiralis* AES could inhibit the DSS-induced recruitment of inflammatory cells into the colon tissue. However, the administration of AES alone also caused limited inflammatory cell infiltration and increased MPO activity in colon compared to PBS control (Fig. 2A, B, C).

### Effect of *T. spiralis* AES on the inflammatory cytokines in mice with DSS-induced colitis

To understand the mechanism that underlies the alleviation of DSS-induced colitis in mice after treatment with the *T. spiralis* AES, we examined the levels of some typical Th1 or pro-inflammatory cytokines (IFN-γ and IL-6), Th2 cytokines (IL-4 and IL-13), a Th17 cytokine (IL-17) and regulatory cytokines (IL-10 and TGF-β) secreted by spleen, MLN and colon lymphocytes from differently treated mice using ELISPOT or ELISA.

**Figure 3:**
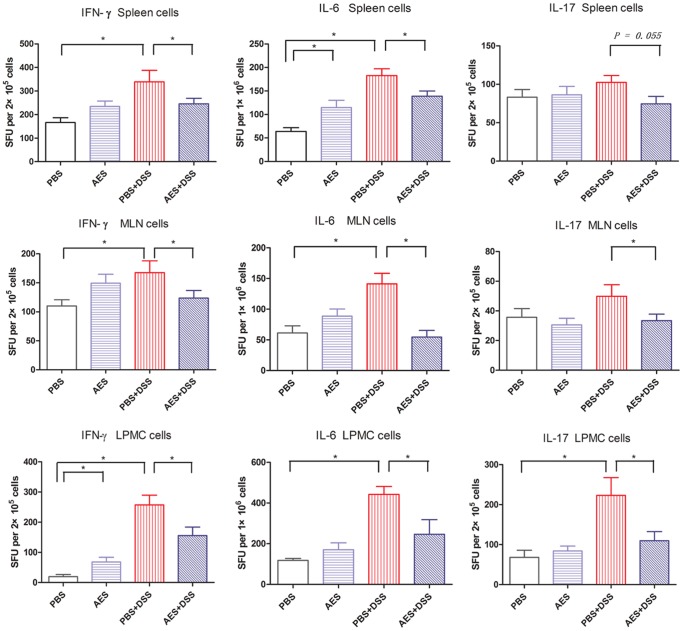
Treatment with the *T. spiralis* AES reduced the levels of the DSS-induced pro-inflammatory cytokines IFN-γ, IL-6 and IL-17 in the spleens, MLN and colon lymphocytes. The data are presented as the mean ± SE. The asterisks* indicate statistical significance at *P<0.05*; SFU, spot-forming units; for spleens and MLN, n = 16 in each group (for IL-17, n = 12); for LPMC, n = 15 in each group (3 mice LPMC pooled).

Compared to the non-treated group (PBS), the levels of the pro-inflammatory Th1 cytokines such as IFN-γ and IL-6 were significantly increased in the spleens, MLN and colon of the mice treated with DSS (PBS-DSS) (*P<0.05*). Similarly, Th17 cytokine level was strikingly increased in the colons of the DSS-receiving groups, but the increase of IL-17 in spleens and MLN of the DSS-induced mice was not statistically significant compared to the non-treated mice (Fig. 3). Treatment with *T. spiralis* AES during the DSS induction (AES-DSS) significantly reduced the spleens, MLN and colon secreted pro-inflammatory cytokines IFN-γ and IL-6 compared to non-treated control group (PBS-DSS) (*P<0.05*). The IL-17 level was also significantly down-regulated by treatment with the *T. spiralis* AES in the colon and MLN of the mice with DSS-induced colitis (Fig. 3).

**Figure 4:**
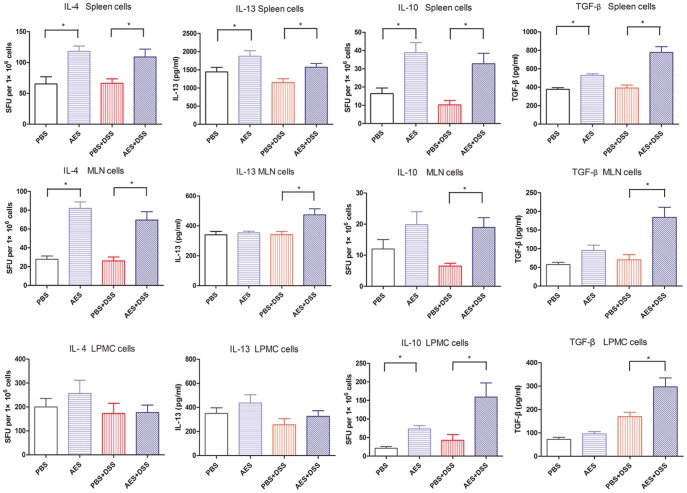
*T. spiralis* AES treatment increased the levels of the anti-inflammatory cytokines IL-4, IL-13, IL-10 and TGF-β in the spleens, MLN and colon lymphocytes. The data are presented as the mean ± SE. Significant differences (*P<0.05*) are indicated as asterisks between groups; SFU, spot-forming units; for spleens and MLN, n = 16 in each group (for IL-13, n = 12); for LPMC, n = 15 in each group (3 mice LPMC pooled).

During investigations of the anti-inflammatory cytokines and Treg response in the treated mice, as shown in Figure 4, we observed a significant up-regulation of IL-10 and TGF-β in the spleens, MLN and colon treated with the *T. spiralis* AES following DSS administration (AES-DSS) compared to group treated with PBS only (PBS-DSS) (*P<0.05*). Interestingly, the increased secretion of IL-10 and TGF-β was also observed in lymphocytes from the colon, MLN and spleen of the control mice treated with AES only (AES) (Fig. 4). These results indicate that the up-regulation of Treg cytokines was mainly induced by the AES and not by the DSS.

Secretion of IL-4 and IL-13 was significantly increased in MLN and spleen lymphocytes treated with the *T. spiralis* AES alone (AES) or following DSS administration (AES-DSS) compared to group treated with PBS only (PBS-DSS) (*P<0.05*). However, such an increased Th2 response was not observed in lymphocytes isolated from colon tissue (Fig. 4).

These results demonstrate that *T. spiralis* AES down-regulate the lymphocyte produced Th1/Th17 pro-inflammatory cytokines induced by DSS, while up-regulating the regulatory cytokines and partially the Th2 cytokines.

### AES treatment induces the generation of Tregs in the MLN tissue

**Figure 5:**
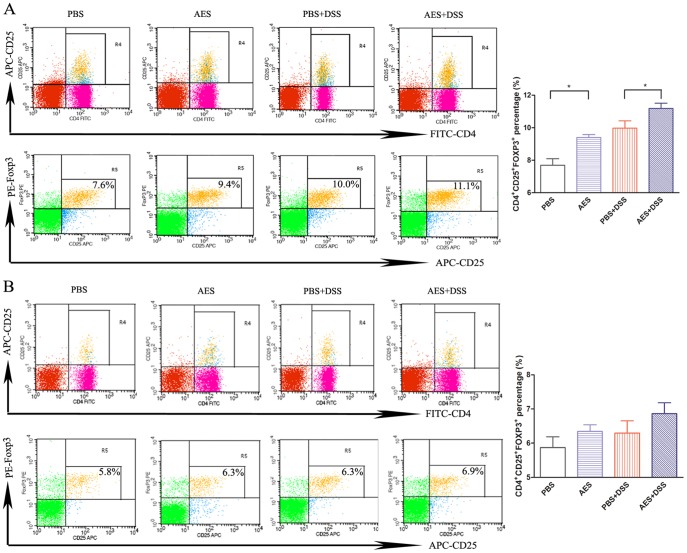
Analysis of the Tregs in the MLN and spleen lymphocytes of mice treated with DSS and *T. spiralis* AES. The lymphocytes isolated from the MLN (A) and spleen (B) of the treated mice were stained with fluorescently conjugated mouse mAbs (anti-CD3e, CD4 and CD25), and the intracellular expression of Foxp3 was observed. Representative data from the FACS analysis of the CD4^+^CD25^+^FOXP3^+^ Tregs are shown on the left with the percentage of double-positive cells in the right corner. The corresponding bar graphs are shown on the right and indicate the mean percentage ± SE. Significant differences are indicated as asterisks (*P<0.05*) compared to the PBS-treated control group; n = 12 (number of mice included in each group).

To further examine if treatment with *T. spiralis* AES stimulates the T-regulatory cells, the surface expression of CD3e, CD4 and CD25 and the intracellular expression of Foxp3, the most accepted marker of Tregs, were examined on lymphocytes isolated from the spleens and MLN of the mice treated with DSS and/or *T. spiralis* AES by FACS. As shown in Figure 5A, the CD3e^+^CD4^+^CD25^+^FOXP3^+^ Tregs were significantly induced in the MLN cells from the mice with DSS-induced colitis followed with *T. spiralis* AES treatment compared to the DSS-induced mice (*P<0.05*). In addition, the Tregs were also significantly increased in the MLN lymphocytes of the mice treated with AES alone compared to the PBS control group (*P<0.05*). However, the DSS-treated mice also displayed increased CD3e^+^CD4^+^CD25^+^FOXP3^+^ Tregs even though the percentage was significant lower compared to the group followed with AES treatment. It is possible to observe increased Tregs for autoimmune diseases; however, the functions of the Tregs in these diseases are typically impaired, and the impaired Tregs are usually unable to regulate the excessive immunopathology [52].

Although not significant, *T. spiralis* AES also tend to augment the percentage of the Tregs cell subset in the spleens of mice with DSS-induced colitis (*P =  0.19*) (Fig. 5B). Apparently, these results, combined with the cytokine profile, suggest that the *T. spiralis* AES treatment could induce the generation of Tregs in mice to suppress the inflammatory response induced by the DSS.

## Discussion

Abundant evidence has demonstrated that helminth infections have the ability to ameliorate IBD [20], [53]. Elliott et al. [54] were the first to propose using helminthic parasites as a practical treatment for Crohn's disease. The preventive and therapeutic effects of different helminth species and developmental stages on experimental colitis were then investigated [55], [56], [57], [58]. Treatment with nematode, trematode or cestode helminths or their products reduced the severity of colitis in different animal models [38], [59].

*Trichinella* infections have been found to down-regulate the inflammatory immunopathology caused by autoimmune diseases in various animal models such as experimental autoimmune encephalomyelitis (EAE) in rats [11], [43], [60], [61] and experimental colitis in mice [38], [39]. However, the mechanism or the molecules involved in this immunomodulation are not quite known. As is known, the *Trichinella* life cycle has three stages, including the intestinal adult worm and the muscle larval stage, which may directly exert an immunomodulatory effect on the host immune response through releasing soluble ES proteins. The ES proteins from *T. spiralis* muscle larvae (MES) have shown to be able to stimulate bone-marrow derived dendritic cells (DCs) to produce a mixed Th1/Th2 cytokine profile with the predominance of Th2 and regulatory cytokines [29]. Mice received *T. spiralis* MES simulated DCs significantly boosted Treg cells that secreted regulatory IL-10 and TGF-β cytokines [62], and decreased production of IFN-γ and IL-17 that leaded to the amelioration of experimental autoimmune encephalomyelitis [60], [61]. Except for MES, *Trichinella* AES was also able to inhibit pro-inflammatory cytokines and induce regulatory cytokines such as IL-10 and TGF-β via macrophage cells *in vitro* [29], [32], suggesting AES also plays similar roles in regulating immunological disorders. In the present study, we generated ES products from *T. spiralis* adult worms and applied them to mice with DSS-induced colitis to investigate whether *T. spiralis* adult worm-derived protein components have therapeutic effects on relieving DSS-induced colitis inflammation.

Here, we demonstrate for the first time that ES products from adult *T. spiralis* (AES) can alleviate acute inflammatory colonic disease in mice induced by DSS. The reduced severity of the disease after AES treatment was associated with an inhibition of Th1/Th17 pro-inflammatory cytokine and an increase in the production of anti-inflammatory Th2/Treg regulatory cytokines in the lymphocytes of colon, MLN and spleen of treated mice. Furthermore, we identified that Treg cells was significantly increased in the MLN of the AES-treated mice compared to the non-treated control mice. The Treg cells were also increased in the spleen of the AES-treated mice although not high enough to be statistically significant, possibly because the Treg response happens first in the proximal end of the lymph node (MLN) to the inflammatory intestine. The Treg cells were not directly detected in the local colon tissue due to the difficulty to retrieve enough lymphocytes to apply intracellular staining even though the level of IL-10 and TGF-β was found to be significantly increased in the colon.

The DSS-induced experimental colitis in mice exhibits many of the symptoms observed in human IBD [63], including diarrhea, bloody feces, mucosal ulceration, and weight loss. This inflammatory process was also accompanied by colon inflammatory cell infiltration and an increase in the MPO activity of the mice [44]. In this study, we demonstrated that treatment with *T. spiralis* AES significantly reduced overall disease manifestations and inflammatory parameters induced by DSS, including a reduced DAI score, attenuated changes in colon length, a decrease in the macroscopic and microscopic inflammation score, and a decrease in the intestinal MPO activity. The reduction in neutrophils influx and MPO activity after *T. spiralis* AES treatment observed in this study was associated with a decrease in colon damage induced by DSS, suggesting that the *T. spiralis* adult worm secreted proteins may have direct inhibition on the neutrophil activities. Indeed, Bruschi et al [64] previously described a 45 kDa glycoptotein secreted by *T. spiralis* significantly inhibited human neutrophil migrations *in vitro* and reduced the up-regulation of the CD11b induced by formyl-methionyl- leucyl-phenylalanine (f-MLP), suggesting an anti-inflammatory function. Similar inhibition on MPO activity was also reported in colitis treated with *T. spiralis* infection [38].

Previous studies mainly focused on muscle larvae developmental stage and indicate that *T. spiralis* muscle larvae crude antigen or recombinant p53 (main component of MES) are able to alleviate colitis in mice [59], [65]. Our study indicated that excretory/secretory products from *T. spiralis* adult worms could also be effective candidates in regulating inflammatory colitis. Since *Trichinella* worms possess a unique stichosome - a modified esophageal gland that contains single-layered stichocyte cells [66]. The stichosome produces rich ES antigens that are released through the anterior ends of adult worms embedded in the intestinal mucosa. This specific structure and secreted ES antigens of adult worm may play important roles in altering host cell physiology and modifying host immune response [67]. In this study, we have demonstrated that the beneficial effects of immune disorder treatments using living worms can be replaced with the ES proteins derived from adult *Trichinella* worms. The major mechanism for the helminth-secreted proteins involved in the host immunomodulation is believed to be an up-regulation of the regulatory T lymphocytes (Tregs) that produce more regulatory cytokines, such as IL-10 and TGF-β, to inhibit the Th1 and Th17-promoted inflammation. It benefits the helminths to survive the host Th1-mediated cellular attack, simultaneously preventing the host's immune system from overreacting to innate or external immunogens or allergens [7], [23]. Th17 is another subset of CD4 T cells that produce IL-17, a pro-inflammatory cytokine involved in the autoimmune diseases and other immunopathology [61], [68]. In this study, we have observed the high level of pro-inflammatory cytokines (IFN-γ, IL-6 and IL-17) produced by lymphocytes from spleen, MLN or colon of mice with DSS-induced colitis, further suggesting these pro-inflammatory cytokines are involved in the inflammation of colitis. The IL-6 also contributes to the differentiation of Th17 cells, aggravating immunopathogenesis of IBD [69]. After being treated with *T. spiralis* AES, the Th1 and Th17 cytokines were significantly reduced, simultaneously correlating with the boost of Treg cells and their produced regulatory cytokines (IL-10 and TGF-β). An increased IL-10 level was also detected in patients infected with *Schistosome*, who displayed a less severe skin-prick test to allergen [70]. Mice with IL-10 knocked out developed more severe lupus, an autoimmune disease mediated by pathogenic Th1 cytokine responses [71]. These results have led to the suggestion that the IL-10 and/or TGF-β secreted by Tregs in response to a chronic helminth infection or helminth-secreted molecules directly moderate Th1-mediated immunopathology. In this study, increased Treg cells as well as the higher level of their secreted IL-10/TGF-β, and decreased pro-inflammatory cytokines including Th1 and Th17, were observed in DSS-induced colitis mice treated with *T. spiralis* AES, further suggesting the immunomodulatory effects of *T. spiralis* AES and its pharmaceutical potential for the treatment of autoimmune or allergic diseases.

Helminthic infections or worm-derived products typically induce strong Th2 immune response which contributes to the protective immunity [27], [29]. The Th2 cytokines IL-4 and IL-13 were apparently boosted in MLN and spleen lymphocytes of mice treated with *T. spiralis* AES in this study, but not significantly in treated mouse colons that represents a local response to DSS-induced colitis. The Th2 response in colon upon *T. spiralis* AES treatment may be offset by a strong local inflammatory reaction. Ruyssers et al. [25] and Cancado et al. [26] also observed that treatment with helminth proteins did not significantly alter the level of IL-4 in the colon with colitis. In fact, Bodammer et al. [57] have shown soluble egg antigen of *Schistosome* failed to improve colitis even though it induced a robust Th2 response, suggesting that Th2 response may not be essential for the control of colonic inflammation. Therefore, our results suggest that the beneficial effect of *T. spiralis* AES to inflammatory colitis may not be Th2-mediated, but rather act through an overall effect of boosting Treg and restraining Th1/Th17.

In this study we have showed the *T. spiralis* adult secreted proteins (AES) apparently inhibited DSS-induced inflammation mainly through inhibiting Th1 cytokines and stimulating regulatory T-cells, however, AES themselves tend to cause minor inflammation in administrated mice, observed by limited inflammatory cell infiltration and increased MPO activity compared to PBS control (Fig. 2A, B, C). The increases of relevant Th1/Th2 cytokines were also observed in the AES-alone treated mice compared to PBS control except for stimulating Treg cytokines (IL-10 and TGF-β) (Fig. 3, 4). It correlates with the findings that *T. spiralis* derived antigens induced a mixed Th1/Th2 responses through stimulating dendritic cells [29]. It was assumed that *T. spiralis* worms secrete various proteins that play different biological and immunological functions except for the immunomodulatory effects [29], [30], [31]. Therefore, it is important to identify the active components in the complex of the nematode-secreted proteins that contribute only to the immunomodulation of inflammatory and auto-immune diseases. Using the identified active protein(s), rather than the AES complex, for the therapy or prevention of autoimmune diseases is a more feasible approach.

In conclusion, *T. spiralis* adult ES products ameliorate DSS-induced colitis in mice, however, these worm-derived products are difficult to be manufactured on a large-scale, and their complicated components may lead to undesired side-effects or other safety issues. Studies are currently underway to screen for and ultimately to identify the effector molecule(s) from *T. spiralis* AES that play roles in immunomodulatory effects for potential large-scale pharmaceutical applications for allergic or autoimmune diseases.
